# Investigation and analysis of the radiation protection status of radiation workers during the peri-pregnancy period

**DOI:** 10.3389/fpubh.2025.1501027

**Published:** 2025-06-23

**Authors:** Binghua Liang, Xiaojian Ji, Tao Zhang, Xiang Zhou, Ziyao Yu, Jianwei Sun

**Affiliations:** ^1^Department of Radiology, Wuxi Xishan District Traditional Chinese Medicine Hospital, Wuxi, China; ^2^Department of Radiotherapy, Xuzhou Cancer Hospital, Xuzhou, China; ^3^Department of Radiology, Wuxi Xishan People’s Hospital, Wuxi, China

**Keywords:** radiation, practitioners, peri-pregnancy, radiation protection, questionnaire survey

## Abstract

**Objective:**

Radiation exposure during pregnancy poses serious risks to fetal health, including increased likelihood of miscarriage, preterm birth, congenital anomalies and developmental disorders, while also impacting maternal occupational safety. This study aimed to assess the concerns and needs of radiation practitioners regarding radiation exposure during the perinatal period, with a focus on radiation protection.

**Methods:**

A questionnaire survey of 147 radiation practitioners from public and private hospitals was conducted to assess their knowledge, concerns and needs regarding radiation protection during the peri-pregnancy period. Statistical analysis was used to compare the importance and implementation of radiation protection in different groups, and chi-squared tests were used to compare differences in policy implementation (public vs. private hospitals), attitudes toward radiation avoidance (male vs. female practitioners) and concerns about fetal exposure across age groups.

**Results:**

Public hospitals demonstrated higher rates of radiation protection policies (39.37%) than private hospitals (21.43%). Among female respondents, 95.12% advocated for temporary removal from radiation-related positions when preparing for pregnancy. Of those who gave birth while working in radiation fields, 26.53% ceased radiation work pre-pregnancy, 30.61% avoided it post-pregnancy and 42.85% did not avoid it. Additionally, 86.58% of female respondents emphasized the need to avoid radiation work during breastfeeding. Among the male participants, 47.83% expressed concern about radiation effects on their fetuses compared with 90% of the female participants.

**Conclusion:**

Radiation protection measures were more effectively implemented in public hospitals than in other institutions, underscoring the need for standardized policies across all institutions. Female practitioners exhibited heightened concerns about radiation exposure of the fetus and infant, particularly during pregnancy and lactation. Strengthening policies and workplace adjustments are critical to mitigating occupational risks and safeguarding maternal and child health.

## 1 Introduction

Radiotherapy, interventional radiology, nuclear medicine and other technologies – such as computed tomography, fluoroscopy and X-ray diagnostics – have rapidly become indispensable components of modern radiological practice, offering transformative diagnostic and therapeutic solutions for patients (1). However, ionizing radiation is a double-edged sword. Although it provides key benefits in clinical applications, it poses serious occupational risks to healthcare workers and patients, particularly during sensitive biological phases such as pregnancy (2, 3). Radiation exposure during the peri-pregnancy period – defined here as 3 months prior to conception, the entire gestation period and up to 6 months postpartum (including lactation) – can adversely affect fetal health, increasing the likelihood of preterm labor, miscarriage, congenital anomalies and developmental disorders (1, 4). These risks necessitate stringent radiation protection measures tailored to this vulnerable population (5, 6).

In China, radiation safety and protection have always been important issues in occupational health. Numerous studies have emphasized the importance of radiation safety for healthcare workers, particularly during the peri-pregnancy period. Many papers have highlighted the specific risks faced by peri-pregnancy workers exposed to radiation (7, 8) and recommend workplace modifications to reduce radiation injuries. The National Standard of the People’s Republic of China (9) (GB 10252–2009) sets an annual effective dose limit of 5 mSv for radiation workers and 0.1 mSv for the public to ensure radiation exposure remains within safe limits. However, monitoring data from 2014 to 2018 in Jiangxi Province revealed that although most radiation workers’ exposure is well controlled (average dose: 0.316 mSv), a small percentage exceeded 1 mSv annually, with 0.10% surpassing 5 mSv. This indicates heightened exposure risks, which is especially concerning when it affects peri-pregnant women and fetuses (10–12). Internationally, the ‘as low as reasonably achievable’ (ALARA) principle guides radiation safety; however, its implementation for peri-pregnant workers remains inadequate (13). Key gaps include the absence of pregnancy-specific dose constraints, inconsistent protocols for temporary job adjustments and limited guidance on fetal dose monitoring. For example, although ALARA emphasizes minimizing exposure, it does not address the unique physiological vulnerabilities of pregnant workers or provide actionable steps for institutions to reconfigure high-risk tasks. Although the International Commission on Radiological Protection (ICRP) provides general guidance, including the ALARA principle, implementation lacks specific criteria tailored to peri-pregnancy protections. Current standards fail to address the unique needs of peri-pregnant radiation practitioners, constituting a gap in clear, actionable guidelines for this vulnerable group (14). It is crucial to better understand and improve the radiation protection status of radiation workers during pregnancy.

This study investigates the radiation protection status of workers during the peri-pregnancy period, focusing on their awareness, workplace practices and unmet needs. By identifying systemic shortcomings and proposing evidence-based solutions, this research aims to bridge the gap between generic safety guidelines and the specific protections required for peri-pregnant practitioners, ultimately safeguarding maternal and fetal health while maintaining occupational efficiency.

## 2 Methods

### 2.1 Study population

The sample size was determined using Kendall’s sample estimation method (15), which is particularly suitable for exploratory studies with multiple variables, ensuring that the sample size (5–10 times the number of variables) adequately captures variability while maintaining feasibility. With 17 variables in the questionnaire, a sample size of 85–170 was targeted. A total of 147 radiation practitioners were recruited via convenience sampling from public hospitals (*n* = 127), private hospitals (*n* = 14) and other institutions (*n* = 6) in China.

The 147 radiation practitioners included radiologists, radiographers, radiology nurses and other radiation-related medical staff from three types of institutions: public hospitals, private hospitals and radiation technology departments of other enterprises and institutions (e.g., radiotherapy, imaging, nuclear medicine and radiology departments). The survey was conducted over 6 months between 20 November 2021 and 20 May 2022.

The inclusion criteria were as follows: (1) full-time employment as a radiation practitioner (including radiologists, radiographers, radiology nurses and nuclear medicine staff); (2) active engagement in radiation-related duties during the study period (November 2021–May 2022); (3) aged between 20 and 50 years, covering the primary reproductive age group; and (4) ability to communicate independently and provide informed consent.

The exclusion criteria were as follows: (1) part-time workers or temporary staff, to ensure consistency in occupational exposure assessment; (2) individuals on maternity leave, sick leave or extended absence during the study period; (3) practitioners who had permanently left radiation work due to pregnancy or health concerns prior to the study; (4) cognitive impairment, neurological disorders or severe physical/mental health conditions affecting questionnaire completion; and (5) refusal to provide informed consent or incomplete responses to >20% of the questionnaire items.

This study was approved by the Ethics Committee of Xuzhou Cancer Hospital (Approval No. 2021–02-016-K01). Written informed consent was obtained from all participants.

### 2.2 Questionnaire design

A narrative review of national and international regulations was conducted to inform the questionnaire design. Key documents included China’s Health Standards for Radiation Protection (GB 10252–2009), ICRP guidelines and peer-reviewed studies on occupational radiation exposure. The preliminary questionnaire was pretested with 15 radiation practitioners (with intermediate professional titles) to assess clarity, relevance and feasibility. Based on their feedback, ambiguous terms (e.g., ‘accelerator scattering’) were rephrased, overlapping questions were consolidated and a Likert scale was added to quantify concern levels. Three radiation protection experts independently reviewed the questionnaire to ensure alignment with national standards (GB 10252–2009). The pretest participants confirmed that the questions were comprehensible and relevant to their work experiences. Although formal statistical reliability testing (e.g., Cronbach’s alpha) was not performed due to the exploratory nature of the study, internal consistency was assessed qualitatively. Participants’ responses to related questions showed logical coherence, supporting the questionnaire’s reliability.

The final survey, titled Survey on Radiation Protection of Practitioners During the Peri-Pregnancy Period (Supplementary material 1), comprised 17 questions organized into four domains, as follows:

(1) Demographics: institution type, age and gender.
(2) Workplace practices: radiation sources, dosimeter readings and policy awareness.
(3) Peri-pregnancy protections: preconception adjustments, lactation precautions and fetal health concerns.
(4) Health outcomes: miscarriage, preterm labor and birth defects.

### 2.3 Statistical analysis

A total of 159 questionnaires were distributed, and 147 were returned. The survey results were processed using the statistical screening function of the Questionnaire Star platform, and the chi-squared test was performed using cross-tabulation in SPSS 26.0 to analyze differences between groups regarding their level of concern for radiation protection. The significance level for the test was set at *α* = 0.05.

## 3 Results

### 3.1 General information

**Figure 1:**
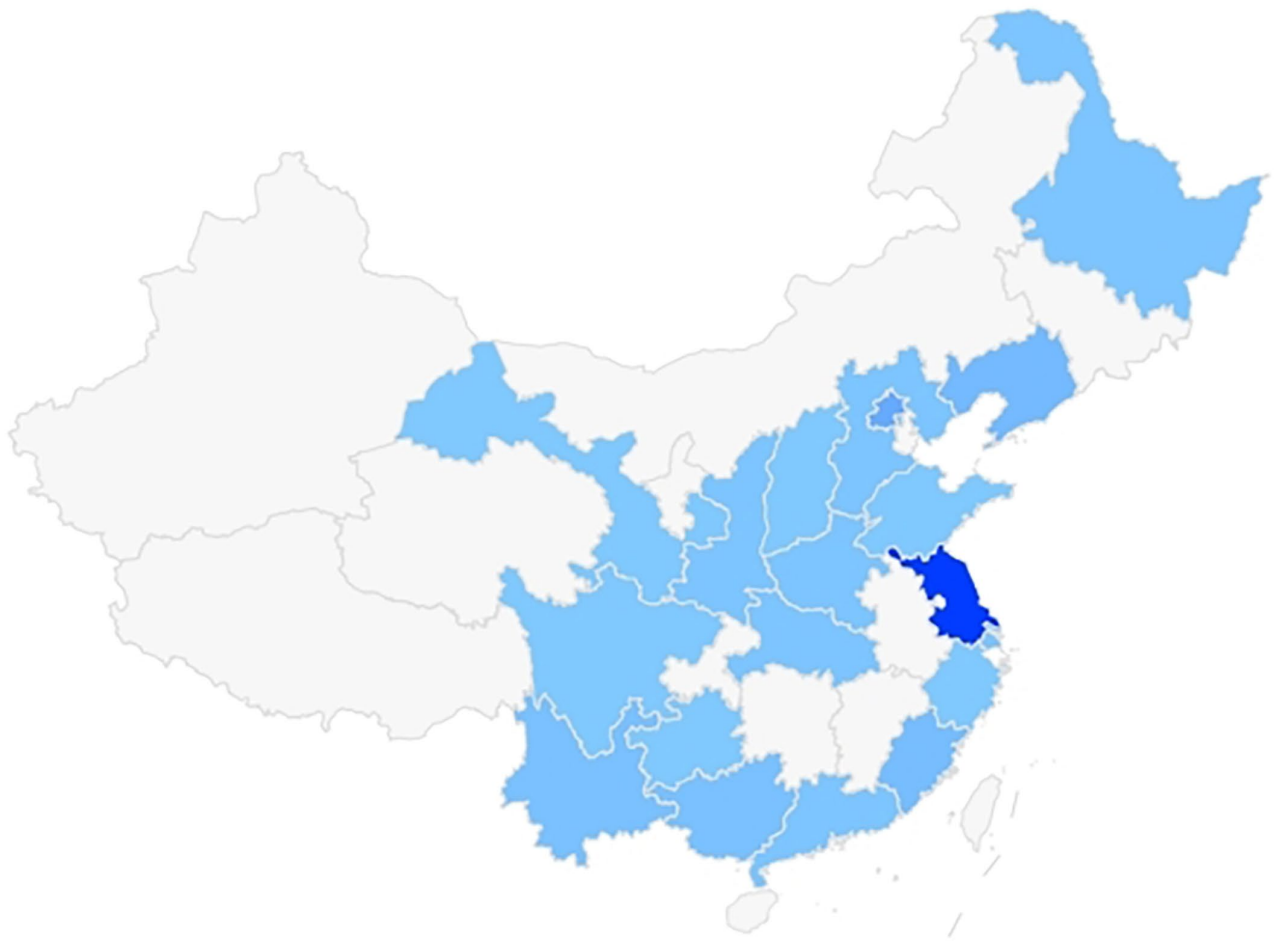
The distribution of participants’ locations.

A total of 147 radiation practitioners participated, predominantly from public hospitals (86.40%, *n* = 127), with 55.78% (*n* = 82) being women and 44.22% (*n* = 65) men (Table 1). The majority (70.06%, *n* = 103) were aged 31–40 years, reflecting the core workforce in radiation-related roles (Table 2). The distribution of participants’ locations was relatively widespread, as shown in Figure 1.

**Table 1:** Demographic distribution of participants by institution type and gender (*n* = 147).

Item	Respondents (Count)	Respondents (Percentage)
Affiliation		
From public hospitals	127	86.40%
From private hospitals	14	9.52%
From other enterprises and institutions	6	4.08%
Gender distribution		
Male	65	44.22%
Female	82	55.78%
Peri-pregnancy radiation protection policies		
Public hospitals	50/127	39.37%
Private hospitals	3/14	21.43%
Other enterprises and institutions	0/6	0.00%

**Table 2:** Age distribution and understanding of radiation protection laws among radiation workers.

Item	Respondents (Count)	Respondents (Percentage)
Age		
20–30 years	30	20.41%
31–40 years	103	70.06%
41–50 years	10	6.81%
> 50 years	4	2.72%
Level of knowledge on radiation protection laws		
Basic understanding	108	73.47%
Good understanding	23	15.65%
No knowledge	16	10.88%

### 3.2 Presence of radiation protection policies in hospitals of different types

Public hospitals exhibited higher rates of peri-pregnancy radiation protection policies (39.37%) compared with private hospitals (21.43%) (Table 1). However, <40% of public hospitals had formalized regulations, indicating systemic gaps across all institutions.

### 3.3 Degree of understanding of radiation protection laws and regulations among radiation workers of different ages

The respondents in this survey were divided into four age groups: 20.41% (*n* = 30) were aged 20–30 years, 70.06% (*n* = 103) were aged 31–40 years, 6.81% (*n* = 10) were aged 41–50 years and 2.72% (*n* = 4) were aged >50 years. Regarding their understanding of radiation protection laws and regulations, 73.47% (*n* = 108) reported having a basic understanding, 15.65% (*n* = 23) indicated a very good understanding and 10.88% (*n* = 16) stated they did not know the relevant laws and regulations (Table 2).

### 3.4 Radiation sources in the working environment of radiation practitioners and the cumulative annual effective dose

Accelerator scattering (79.52%) and imaging equipment (65.06%) were the primary radiation sources. Only 7.23% reported annual doses >1 mSv, and 25.3% were unsure of their exposure levels (Table 3).

**Table 3:** Knowledge of radiation protection and work environment information.

Question	Option	Percentage
Radiation sources in work environment (Multiple Choice)	Accelerator scattering	79.52%
	Imaging equipment scattering	65.06%
	Patient radiation from implants	21.69%
	Leakage from lead doors	56.63%
	Others	16.87%
Annual dose on personal dosimeter > 1 mSv	Yes	7.23%
	No	67.47%
	Do not know	25.3%
Need to avoid radiation before pregnancy	Necessary	87.95%
	Not necessary	12.05%

### 3.5 Gender and age differences in radiation avoidance attitudes

Female practitioners were significantly more likely to advocate for temporary removal from radiation work when preparing for pregnancy (95.12% vs. 78.46% of men). Among those who gave birth while working in radiation fields, only 26.53% (*n* = 39) left their positions pre-pregnancy, whereas 42.85% (*n* = 63) did not avoid radiation exposure at all. Concerns about radiation exposure during lactation were higher among women (86.58% vs. 63.08% of men) (Table 4). Among the male participants, 47.83% expressed concern about radiation effects on their fetuses compared with 90% of the female participants, indicating that female employees were significantly more concerned than their male counterparts.

**Table 4:** Investigation results of whether the pregnancy is free from radiation environment.

Options	Respondents (Count)	Respondents (Percentage)
Get out of radiation work before pregnancy	39	26.53%
Get out of radiation work after pregnancy	45	30. 61%
Not leaving the radiation position	63	42. 85%
Attitude toward leaving workplace to prepare for pregnancy (Male)	51	78.46%
Attitude toward leaving workplace to prepare for pregnancy (Female)	78	95.12%
Attitude toward breastfeeding women leaving radiation work (Male)	41	63.08%
Attitude toward breastfeeding women leaving radiation work (Female)	71	86.58%

Additionally, concerns about the fetal effects of radiation varied significantly across age groups. Younger practitioners (20–30 years) universally expressed concerns about fetal radiation effects (100%) compared with 65.63% of those aged 31–40 and 50% of those aged ≥41 years (*p* = 0.003) (Table 5).

**Table 5:** Investigation results of whether the fetus is worried about being affected by radiation.

Practitioners	Respondents (Percentage)	*p* value
Male	47.83%	0.001
Female	90.00%	
aged ≥41 years	50.00%	0.003
aged 31–40 years	65.63%	
aged 20–30 years	100.00%	

### 3.6 Responses to open-ended questions

The last open-ended question (‘What suggestions do you have for radiation management during peri-pregnancy among radiation practitioners?’) resulted in 20 suggestions, which were categorized into five themes:

(1) Policy reforms

- Mandate temporary transfers for peri-pregnancy workers (*n* = 6).
- Establish legal protections against wage deductions during pregnancy (*n* = 3).

(2) Workplace adjustments

- Implement remote operation technologies (*n* = 4).
- Prohibit pregnant workers from high-exposure tasks (*n* = 2).

(3) Health monitoring

- Conduct longitudinal studies on fetal health outcomes (*n* = 3).
- Provide regular health assessments for pregnant workers (*n* = 2).

(4) Training initiatives

- Enhance dosimeter usage training (*n* = 3).
- Offer public education on radiation risks (*n* = 2).

(5) Financial support

- Provide paid radiation leave (*n* = 4).
- Provide subsidies for non-transferable roles (*n* = 2).

## 4 Discussion

### 4.1 Policy implementation gaps across institutions

Recent studies have continued to highlight the complexities of radiation protection, particularly during sensitive periods such as pregnancy (16–19). Female practitioners exhibited significantly higher concern about fetal radiation effects (90% vs. 47.83% of men, *p* < 0.001), likely influenced by direct maternal responsibility (18). Younger workers (aged 20–30 years) universally expressed concerns (100%), possibly due to heightened awareness of prenatal care, whereas older cohorts (≥41 years) showed reduced vigilance (50%). These trends highlight the need for age-tailored education programs (19, 20).

Several notable issues were identified in this study. The survey results indicate that 98.8% of the respondents believed clear industry standards were essential for protecting radiation workers during the peri-pregnancy period. However, as society evolves, existing industry standards reveal certain shortcomings, and relevant departments are actively working to improve and coordinate these regulations. For example, the survey showed that only 38.55% of the units had policies in place to protect radiation workers during the peri-pregnancy period. Employees from units without such regulations expressed a desire for the implementation of protective measures to better safeguard the personal and reproductive health of radiation workers, particularly during the peri-pregnancy period.

### 4.2 Compliance with dose limits and future directions

Despite the national standards of the People’s Republic of China and the principle of optimal protection (≤0.1 mSv for the public), 7.23% of practitioners exceeded 1 mSv annually, echoing findings obtained by Kong et al. (21). This aligns with findings obtained by Almén and Mattsson (7), who identified inconsistent policy adoption as a global challenge. The lack of standardized protocols exacerbates exposure risks, particularly for peri-pregnant workers. Unskilled dosimeter usage, as noted in studies (22–25), further complicates exposure monitoring. Future research should prioritize multicentre studies to validate these trends and explore psychosocial impacts through validated stress scales. Therefore, based on industry statistical data, to comply with protective regulations, reduce exposure risks and alleviate psychological stress, it is recommended that radiation practitioners leave radiation work positions during the peri-pregnancy period.

### 4.3 Bridging the awareness–behavior divide

This study reveals a critical gap between awareness of radiation risks and actionable protective behaviors. Although our questionnaire did not directly measure psychological outcomes using validated scales, several findings indirectly reflect the psychological burden faced by peri-pregnant radiation workers. First, the gender disparity in concerns about fetal radiation effects (90% of women vs. 47.83% of men) suggests that maternal responsibility amplifies anxiety, aligning with studies linking direct caregiving roles to heightened occupational stress (18, 26). This discrepancy suggests that structural barriers, such as fear of career stagnation or lack of alternative roles, may override personal safety concerns, amplifying psychological stress. Second, the universal concern among younger practitioners may stem from heightened prenatal care awareness coupled with fears of career repercussions, a phenomenon noted in oncology nursing literature (26). Third, open-ended responses revealed demands for policy reforms and workplace adjustments, which implicitly signal distress over balancing occupational duties and perinatal safety. These findings align with oncology nursing studies, where role inflexibility and financial pressures exacerbated anxiety among pregnant workers (27). Beyond physical protection, psychological counseling and health monitoring have gained increased attention. Implementing positive psychological support mechanisms and conducting regular health examinations can alleviate the anxiety and stress experienced by pregnant workers, thereby enhancing overall occupational health and safety (20).

Public hospitals demonstrated marginally better radiation protection policies (39.37%) than private hospitals (21.43%); however, <40% of institutions had formalized guidelines. This gap is attributed to the following factors: systemic barriers, such as job security concerns, where fear of demotion or reduced career opportunities deters temporary leave; role inflexibility, characterized by a lack of non-radiation roles that forces practitioners to continue with high-exposure tasks; and financial pressures, including the absence of paid leave or subsidies that compels continued work despite potential health risks. These challenges are reminiscent of those reported in oncology nursing studies, highlighting the urgent need for institutional support mechanisms to address these issues.

Liu et al. (26) noted that the protection principle of occupational radiation for pregnant women states that, as a member of the public, the fetus should receive roughly the same protection as the public, which is consistent with the viewpoint of this article. Wang and Zhu (27) found that approximately 23.33% of nurses in radiotherapy departments experienced symptoms of possible abortion, 6.67% had stillbirths and 3.33% had congenital heart disease, highlighting the prevalence and severity of the issue. Additionally, Huang Li conducted a survey on occupational protection for nurses in oncology radiotherapy and chemotherapy departments during pregnancy, and Wang Ping (28) surveyed the current status of occupational protection for pregnant nurses in operating rooms. Both studies proposed various management strategies to enhance protection for pregnant nurses in these high-risk environments.

Based on the survey results and the current research, this study explores multiple perspectives and proposes the following targeted strategies to better safeguard the health and rights of peri-pregnancy radiation practitioners.

1. Improve industry standards and policies: The government and industry associations should accelerate coordination to ensure the formulation and implementation of specific and operational industry standards for the protection of perinatal practitioners.
2. Strengthen internal rules and regulations of the unit: All medical units (especially private hospitals) should be encouraged to formulate clear peri-pregnancy radiation protection policies and provide corresponding training and resources to help practitioners reduce radiation exposure during pregnancy planning. Appropriate radiation protection practice training must be conducted for the staff, especially during the perinatal, pregnancy and breastfeeding periods.
3. Improve the working environment and processes: The radiation exposure of workers should be reduced through technology and operational processes, such as using remote operation and automated equipment.
4. Psychological support and health assessment: Psychological counseling and support services should be provided to help alleviate the psychological stress of peri-pregnancy workers, health monitoring measures should be strengthened, regular physical examinations and health assessments conducted and health problems identified and addressed promptly.
5. Expand and deepen research: Larger-scale surveys and studies should be conducted, particularly focusing on private hospitals, distinguishing the risk differences between departments to address existing data deficiencies and encouraging multicentre research to share and integrate the experiences and results of various regions and units to develop unified, widely applicable policy recommendations.
6. Enhance public and practitioner awareness: Educational initiatives should be promoted to improve the knowledge and awareness of radiation protection among practitioners, enabling their active participation in occupational health management. Scientific research should be encouraged to validate the effectiveness of current protection measures and foster the improvement and widespread adoption of innovative protection technologies.

Through implementing these targeted strategies, occupational safety and health protection for peri-pregnancy radiation workers can be effectively improved and work-related stress can be reduced. By improving the working conditions of peri-pregnancy staff, reducing radiation exposure and enhancing radiation protection measures, fetal health can be safeguarded and the risks of premature delivery, miscarriage and developmental disorders can be minimized.

This study has some limitations. First, private hospitals accounted for a relatively small proportion of the questionnaires received in this survey. Therefore, the conclusion that public hospitals have substantially higher proportions of relevant policies than private hospitals requires further validation with an expanded sample size. Second, the questionnaire did not include a selection for departments, making it impossible to distinguish differences in annual personal cumulative radiation doses across various departments and positions. Designing a supplementary questionnaire could address these limitations and enhance the accuracy and comprehensiveness of the survey findings. We also acknowledge that the absence of direct psychometric measurements limits our ability to link radiation risks to psychological outcomes conclusively. Future studies should integrate validated tools to assess stress, anxiety and coping mechanisms, particularly in cohorts with uncertain radiation exposure.

## 5 Conclusion

This study highlights critical gaps in radiation protection for peri-pregnant workers, with public hospitals marginally outperforming private institutions. Key findings include gender-driven risk perceptions, systemic barriers to protective actions and inconsistent policy implementation. By prioritizing relevant measures, institutions can mitigate occupational hazards, align with global safety standards and safeguard maternal and fetal health.

## Data Availability

The original contributions presented in the study are included in the article/Supplementary material, further inquiries can be directed to the corresponding author.

## References

[1] Valentin JE. The 2007 recommendations of the international commission on radiological protection. ICRP publication 103. Ann ICRP. (2007) 37:1–332. doi: 10.1016/j.icrp.2007.10.003, PMID: 18082557

[2] Wang H Dang S Zeng L Li Q Wang Q Zhao Y. Effects of exposure to special risk factors during the peri-pregnancy period on birth defects in newborns. J Xi’an Jiaotong Univ. (2017) 38:326–31. doi: 10.7652/jdyxb201703002

[3] Katsonouri A Gabriel C Esteban López M Namorado S Halldorsson TI Snoj Tratnik J. HBM4EU-MOM: prenatal methylmercury-exposure control in five countries through suitable dietary advice for pregnancy - study design and characteristics of participants. Int J Hyg Environ Health. (2023) 252:114213. doi: 10.1016/j.ijheh.2023.114213, PMID: 37393843

[4] De Santis M Cesari E Nobili E Straface G Cavaliere AF Caruso A. Radiation effects on development. Birth Defects Res C Embryo Today. (2007) 81:177–82. doi: 10.1002/bdrc.20099, PMID: 17963274

[5] Guan H Shang L. Current situation and prevention of birth defects. Elect J Develop Med. (2019) 7:1–4. doi: 10.3969/j.issn.2095-5340.2019.01.001

[6] Hogan AH Bellin E Douglas L Levin TL Esteban-Cruciani N. Radiation exposure of premature infants beyond the perinatal period. Hosp Pediatr. (2018) 8:672–8. doi: 10.1542/hpeds.2018-0008, PMID: 30301739 PMC6207094

[7] Almén A Mattsson S. Radiological protection of foetuses and breast-fed children of occupationally exposed women in nuclear medicine - challenges for hospitals. Phys Med. (2017) 43:172–7. doi: 10.1016/j.ejmp.2017.08.010, PMID: 28882410

[8] Chu B Miodownik D Williamson MJ Gao Y St Germain J Dauer LT. Radiological protection for pregnant women at a large academic medical Cancer center. Phys Med. (2017) 43:186–9. doi: 10.1016/j.ejmp.2017.04.012, PMID: 28457788 PMC5659964

[9] Xu B Zhao X Feng Z Li J Liang Y Zhang W. Protocol for evaluating the efficacy and safety of radiotherapy for prostate and Oligometastatic lesions in patients with low-burden sensitive Oligometastatic prostate Cancer: an open, exploratory pilot clinical trial. Cancer Control. (2024) 31:10732748241274595. doi: 10.1177/10732748241274595, PMID: 39180187 PMC11344251

[10] Zhou N Deng L Wang Z Wang J Chen Y Liu Y. Analysis of external exposure personal dose monitoring results of medical radiation workers in Jiangxi Province from 2014 to 2018. Chin J Radiol Med Prot. (2021) 41:116–21. doi: 10.3760/cma.j.issn.0254-5098.2021.02.007

[11] Xiong X. Results and analysis of personal dose monitoring of radiotherapy and nuclear medicine practitioners in Jiangxi Province. Chin J Rad Health. (2006) 15:301–2. doi: 10.3969/j.issn.1004-714X.2006.03.023

[12] Li M Zhang S Jia Y Mai W Liu X Yang Y. Analysis of radiation dose levels of radiotherapy and nuclear medicine workers in Guangdong Province from 2003 to 2012. Chin Occup Med. (2014) 5:527–9. doi: 10.11763/j.issn.2095-2619.2014.05.009

[13] Kaplan DJ Patel JN Liporace FA Yoon RS. Intraoperative radiation safety in orthopaedics: a review of the ALARA (as low as reasonably achievable) principle. Patient Saf Surg. (2016) 10:27. doi: 10.1186/s13037-016-0115-8, PMID: 27999617 PMC5154084

[14] Cousins C Miller DL Bernardi G Rehani MM Schofield P Vañó E. ICRP PUBLICATION 120: radiological protection in cardiology. Ann ICRP. (2013) 42:1–125. doi: 10.1016/j.icrp.2012.09.001, PMID: 23141687

[15] Memon MA Ting H Hwa CJ. Sample size for survey research: review and recommendations. J Appl Struct Equ Model. (2020) 4:1–20. doi: 10.47263/JASEM.4(2)01, PMID: 40236951

[16] Rajaraman P Hauptmann M Bouffler S Wojcik A. Human individual radiation sensitivity and prospects for prediction. Ann ICRP. (2018) 47:126–41. doi: 10.1177/0146645318764091, PMID: 29648458

[17] Applegate KE Rühm W Wojcik A Bourguignon M Brenner A Hamasaki K. Individual response of humans to ionising radiation: governing factors and importance for radiological protection. Radiat Environ Biophys. (2020) 59:185–209. doi: 10.1007/s00411-020-00837-y, PMID: 32146555

[18] Wu YL Christodoulou AG Beumer JH Rigatti LH Fisher R Ross M. Mitigation of fetal radiation injury from mid-gestation Total-body irradiation by maternal Administration of Mitochondrial-Targeted GS-Nitroxide JP4-039. Radiat Res. (2024) 202:565–79. doi: 10.1667/RADE-24-00095.1, PMID: 39074819 PMC11552446

[19] Almohammed HI Elshami W Hamd ZY Abuzaid MM. Enhancing radiation safety awareness and practices among female radiographers: a comprehensive approach. BMC Health Serv Res. (2024) 24:931. doi: 10.1186/s12913-024-11369-2, PMID: 39143457 PMC11325701

[20] Maggen C van Gerwen M Van Calsteren K Vandenbroucke T Amant F. Management of cancer during pregnancy and current evidence of obstetric, neonatal and pediatric outcome: a review article. Int J Gynecol Cancer. (2019) 29:404–16. doi: 10.1136/ijgc-2018-000061, PMID: 30659032

[21] Kong L Chen L Li X Gao Y Wang X. Estimation of radiation dose to patients and radiation workers caused by (18) F-FDG during PET/CT examination. Chin Occup Med. (2017) 44:646–9. doi: 10.11763/j.issn.2095-2619.2017.05.026

[22] Xu W Dai X Zhang J Zhang Y. Analysis of peripheral blood counts of radiation workers in medical institutions in Yangpu District, Shanghai from 2017 to 2019. Shanghai J Prev Med. (2021) 33:833–7. doi: 10.19428/j.cnki.sjpm.2021.20866

[23] Li Y Hou Z. Analysis of radiation dose to the environment and related workers during PET-CT examination of patients with malignant tumors. J Second Mil Med Univ. (2022) 43:229–32. doi: 10.16781/j.CN31-2187/R.20210715

[24] Essop H Kekana M Smuts H Masenge A. Fetal dosimeter access, usage, and training among pregnant radiographers in South Africa. J Radiol Nurs. (2023) 42:496–503. doi: 10.1016/j.jradnu.2023.07.005

[25] Dauer LT Miller DL Schueler B Silberzweig J Balter S Bartal G. Occupational radiation protection of pregnant or potentially pregnant workers in IR: a joint guideline of the Society of Interventional Radiology and the cardiovascular and interventional radiological Society of Europe. J Vasc Interv Radiol. (2015) 26:171–81. doi: 10.1016/j.jvir.2014.11.026, PMID: 25645407

[26] Liu C Shao Y Wang W Jia T. Principles of occupational radiation protection for women. Chin J Radiol Med Protect. (2004) 24:88–9. doi: 10.3760/cma.j.issn.0254-5098.2004.01.041

[27] Wang Z Zhu B. Research progress on occupational protection of radiotherapy nurses during pregnancy. Med Front. (2017) 7:356–7. doi: 10.3969/j.issn.2095-1752.2017.33.311

[28] Wang P. Survey on the current status of occupational protection of pregnant nurses in operating rooms. J Am Acad Orthop Surg. (2020) 15:89. doi: 10.3969/j.issn.1007-8517.2011.01.067

