# Supplementary material for: Investigation and analysis of the radiation protection status of radiation workers during the peri-pregnancy period
Source: Front Public Health. 2025 Jun 23;13:1501027. doi: 10.3389/fpubh.2025.1501027 (PMC12229878; doi:10.3389/fpubh.2025.1501027)
Supplement: Supplementary file 1 [file Supplementary_file_1.docx]

Supplementary Material 1

Investigation on radiation protection of radiation workers during periconceptional period

1. what is your organization? [Single choice] *

| ○Public hospitals |
| --- |
| ○Private hospitals |
| ○Other |

2. your age is? [single choice] *

| ○20-30 years |
| --- |
| ○30-40 years |
| ○40-50 years |
| ○50-60 years |

3. understand the relevant laws and regulations on radiation protection? [Single choice] *

| ○Not understood |
| --- |
| ○Basic understanding |
| ○Very well understood |

4. what are the sources of radiation in your work environment? (multiple choice) [multiple choice] *

| □ Accelerator ray scattering |
| --- |
| □ Imaging equipment ray scattering |
| □ Particle implantation patient radiation |
| □ Jaws leak |
| □ Other |

5. Does your personal dosimeter detect that the annual cumulative dose exceeds 1 msv? [Single choice] *

| ○Yes |
| --- |
| ○No |
| ○Not known |

6. Do you think you need to get out of the radiation post before pregnancy to prepare for pregnancy? [Single choice] *

| ○Required |
| --- |
| ○Not required |

7. your gender: [single choice] *

| ○Male | ○Female |  |  |  |  |  |  |
| --- | --- | --- | --- | --- | --- | --- | --- |

8. have you ever had a child while working on radiation? [Single choice] *

| ○Yes |
| --- |
| ○No (please skip to question 14) |

9. have you (or your loved one) left your radiation post before and during pregnancy? [Single choice] *

| ○Start taking off duty before pregnancy (please skip to Question 14) |
| --- |
| ○Started leaving radiation post after pregnancy |
| ○Not disengaged from radiation post |

10. do you (or your lover) have symptoms of threatened abortion during the first trimester? [Single choice] *

| ○Yes |
| --- |
| ○No |

11. have you (or your loved one) had a premature birth? [Single choice] *

| ○Yes |
| --- |
| ○No |

12. Do you (or your loved one) worry about your baby being affected by radiation during pregnancy? [Single choice] *

| ○Yes |
| --- |
| ○No |

13. Does your child have birth defects? [Single choice] *

| ○Yes |
| --- |
| ○No |

14. do you think you need to get out of the radiation post during lactation? [Single choice] *

| ○Required |
| --- |
| ○Not required |

15. Do you think it is necessary to add clear laws and regulations to protect radiation practitioners during the periconceptional period? [Single choice] *

| ○Required |
| --- |
| ○Not required |

16. does your unit have policies on protecting radiation practitioners around pregnancy? [Single choice] *

| ○Yes |
| --- |
| ○No |

17. What are your suggestions for radiation protection management during the periconceptional period for radiation practitioners? [Fill in blank questions]

_________________________________
